# Eccentric Resistance Training in Youth: A Survey of Perceptions and Current Practices by Strength and Conditioning Coaches

**DOI:** 10.3390/jfmk6010021

**Published:** 2021-02-18

**Authors:** Benjamin Drury, Hannah Clarke, Jason Moran, John F. T. Fernandes, Greg Henry, David G. Behm

**Affiliations:** 1 Department of Applied Sport Sciences, Hartpury University, Gloucestershire GL19 3BE, UK; hannah.clarke@hartpury.ac.uk (H.C.); john.fernandes@hartpury.ac.uk (J.F.T.F.); greg.henry@hartpury.ac.uk (G.H.); 2 School of Sport, Rehabilitation and Exercise Sciences, University of Essex, Colchester CO4 3SQ, UK; jmorana@essex.ac.uk; 3 School of Human Kinetics and Recreation, Memorial University of Newfoundland, St. John’s, NL A1C 5S7, Canada; dbehm@mun.ca

**Keywords:** long-term athletic development, training prescription, injury prevention, strength training, youth athletes

## Abstract

Background: Eccentric resistance training (ERT) in youth is advocated for aiding performance and injury risk. However, research investigating the applied practices of ERT in youth is in its infancy. In this study, we surveyed the perceptions and practices of practitioners utilizing ERT in youth to provide an understanding of its current application in practice. Methods: Sixty-four strength and conditioning coaches completed an online survey reporting their current use of ERT in youth using both open and closed questions. Results: Coaches deemed the inclusion of ERT important in youth with its inclusion based upon factors such as maturation status, training age and strength levels. Coaches also displayed an awareness of the physiological responses to eccentric exercise in youth compared to adults. ERT was primarily used for injury prevention, with the majority of coaches using body-weight and tempo exercises. Furthermore, utilizing eccentric hamstrings exercises was reported as highly important. The frequency of ERT tended to increase in older age groups and coaches mainly prescribed self-selected rest intervals. Finally, the need for further research into the training guidelines of ERT in youth was highlighted, in which coaches require more information on how maturation influences training adaptations and the fatigue–recovery responses. Conclusion: Coaches emphasized the importance of including ERT for both performance and injury prevention factors in youth although further research is required to generate practical guidelines for coaches in order to support its inclusion within practice.

## 1. Introduction

Improving youth athletic performance can be achieved using resistance training (RT) throughout childhood and adolescence [1]. Furthermore, to support long-term athletic development (LTAD), position statements exists pertaining to the safe and effective training prescription of RT [2]. These frameworks provide coaches, working with youth athletes, guidance on how factors such as growth and maturation can influence adaptations to training stimuli as well as how the training process can be structured. Such an approach is necessary considering that performance and adaptive responses in youth are influenced by an individual’s maturity status [3]. This is particularly important with reference to the youth physical development model (YPD) in which the development of muscular strength and power is key [4]. Prior research in youth athletes has reported that higher levels of muscular strength improves performance in tasks such as jumping [5], sprinting [6] and change of direction movements [7] as well as reducing injury risk [8]. Therefore, knowledge of RT methods that can be utilized to enhance physical qualities in youth athletes is important to understand.

One RT method that is commonly utilized to improve athletic performance is eccentric resistance training (ERT). Indeed, the use of ERT has been reported to improve physical qualities such as strength and power [9]. Importantly, improvements following ERT are not necessarily exclusive to the muscle action type with increases in both eccentric, concentric and isometric strength previously shown [10]. Such changes can be explained by neuromuscular [10], morphological [11] and molecular [12] adaptations. Despite this, greater levels of muscle damage and a longer time for neuromuscular function to recover are observed following eccentric exercise due to both central and peripheral factors [13]. Consequently, the incorporation of ERT for athletes has been noted as a challenge by strength and conditioning (S&C) coaches [14]. To support this, practical guidelines have been generated to provide coaches with a framework of how to prescribe ERT for athletes [15]. However, muscular and neural differences exist between youth and adults which influence the response and adaptations to resistance training (RT) [16]. Therefore, the training prescription of ERT for youth athletes is likely to require a more tailored approach.

A breadth of literature currently exists pertaining to the prescription of RT to support LTAD [17,18,19]. Comparatively, little of this information specifically addresses the use of ERT. Although the reasons for this are unclear, previous research has commented that the inclusion of eccentric exercises could be too intense for young and inexperienced athletes [20]. However, the inclusion of RT in youth has been reported to be effective provided that it is age appropriate, safe and supervised [21]. Moreover, potential concerns of male and female youths being at greater risk of fatigue and muscle damage compared to adults following eccentric exercise is not supported by current literature [22,23]. From a performance perspective, ERT in youth has also been shown to lead to improvements in strength and power, change of direction, sprint performance and injury prevention [24,25,26,27,28,29,30,31,32,33]. Additionally, tasks which include high levels of eccentric forces such as jumping, landing, hopping, and deceleration are all considered key athletic motor skills competencies that should be developed in youth [34]. Therefore, whilst the practical application of ERT in youth athletes is still in its infancy, it would appear that its implementation throughout youth has potentially important implications.

To date, conceptual recommendations have been provided with regards to the training prescription of ERT methods in youth [1,35]. However, little is known about its actual implementation within practice for youth athletes. Conversely, the practices of ERT in in elite athletes have been reported [14,36]. Since aspects such as growth and maturation [37] as well as training age [38] influence adaptations to RT it is likely that current practices of ERT in youth will, and should, differ. However, the lack of specific training guidelines available to S&C coaches in this area makes it is unclear what evidence-based approach is currently being undertaken. Consequently, it is important to understand S&C coach’s current knowledge of the area as well as barriers and concerns regarding its inclusion. Such an approach is recommended to allow research to guide practice, but also for practice to guide research [39]. Indeed, the reporting of perceptions and practices of injury prevention strategies by practitioners working with youth athletes has previously highlighted important areas for consideration [40]. Therefore, the purpose of this study was to survey S&C coaches in order to understand their perceptions and current practices of ERT in youth as well as perceived barriers they may face with regards to its inclusion.

## 2. Materials and Methods

### 2.1. Subjects

Coaches were recruited through the use of online platforms (Twitter, LinkedIn and email networks). Inclusion criteria required the strength and conditioning coaches to currently be with youth athletes under the age of 18 y. Informed consent was sought from all coaches prior to completing the questionnaire in which their responses were anonymous. The study was conducted in accordance with the Declaration of Helsinki and by the University Ethics Committee.

### 2.2. Experimental Design

This study used a web-based questionnaire (British Online Surveys, Bristol, UK) to survey the perceptions and current practices of ERT in youth athletes by S&C coaches. The questionnaire included a mixture of open and closed questions which took approximately 15–20 minutes to complete (Supplementary Materials). A total of twelve questions, which included ten closed questions and two open questions, were used from the full completed survey. Questions were framed around four areas including (1) perceptions of ERT in youth; (2) implementation of ERT in youth; (3) training prescription of ERT in youth; and (4) barriers and future directions for the use of ERT in youth. Quantitative responses for closed question responses were primarily provided on a five-point Likert scales to determine perceived importance and extent of agreement. Additionally, several multiple-choice questions were also included requiring either single or multi-response answers. Open questions were used to further understand the coaches concerns and future directions to the inclusion of ERT in youth. ERT was defined as using a load during the eccentric phase that is in excess of the concentric phase [9], whilst traditional resistance training (TRT) was defined as an emphasis on loading the upward concentric phase of an exercise using resistance or body mass [41].

### 2.3. Procedures

An initial survey was designed by a panel of three experts that had both practical and research experience in the topic area. The survey was reviewed for face and content validity [42] via a panel of four experienced S&C coaches currently working with youth athletes. Subsequently, a pilot survey was sent out (*n* = 4) to gain feedback and recommendations on areas of the survey that they believed could be improved. This approach is similar to previous studies that have completed surveys of practitioners within topic areas of eccentric training and youth athletes [36,40]. The panel and pilot survey subjects were from a range of different sports and employment settings within youth to ensure that the questions were appropriate for a wide range of potential subjects that may complete the survey. Once the survey was finalized it was sent out to the target population. Subjects were provided a maximum of six weeks to complete the survey and the lead researcher’s contact details were provided in case any queries or clarity was required regarding the answers to the questions. Subsequently, a total of 64 responses were received for the survey and were included for the analyses.

### 2.4. Statistical Analysis

All data were collected using an online questionnaire (https://www.onlinesurveys.ac.uk/ (accessed on 18 February 2021)). Data were then transferred to Microsoft Excel for further analysis. This observational study followed a descriptive, cross-sectional design, therefore quantitative data presentation is mostly descriptive in nature with frequency counts and percentages calculated. For questions incorporating unipolar Likert scales, responses were coded (e.g., 1 = “least important” or “strongly disagree”, 5 = “most important” or “strongly agree”). Points for each response were then summed to facilitate ranking of highest to lowest in importance [43]. Where possible, for between-group differences in TRT compared to ERT responses a proportion ratio (PR) was calculated in accordance with previous research [44]. The PR magnitudes were calculated and assessed against the following magnitude scale: 1.00, 1.11, 1.43, 2.0, 3.3 and 10 for trivial, small, moderate, large, very large and extremely large, respectively, and their inverses 0.9, 0.7, 0.5, 0.3 and 0.1 [45]. Responses to open questions were sorted into categories for a frequency count by the lead researcher and then discussed with members of the research team to ensure agreement. Areas for future research of ERT in youth were visualized to display the generated themes in accordance with previous research investigating practitioner’s perceptions [46] using WordArt (https://wordart.com/ (accessed on 18 February 2021)).

## 3. Results

### 3.1. Demographic Characteristics of the Coaches

Coaches worked in a variety of youth team sports including rugby union, soccer, Gaelic football, cricket, basketball, swimming, triathlon and weightlifting. Coaches were from the United Kingdom (86%, *n* = 55), United States of America (3%, *n* = 2), Portugal (3% *n* = 2), Sweden (1.5%, *n* = 1), France (1.5%, *n* = 1) and Canada (1.5%, *n* = 1) with the remaining coaches not reporting this information (3%, *n* = 2). Overall, 76% (*n* = 49) of coaches worked in professional sport, 14% (*n* = 9) in schools/colleges and 10% (*n* = 6) in semi-professional sport. With regard to the sexes that the coaches coached, 78% (*n* = 50) worked with male youth athletes exclusively, 2% (*n* = 1) worked with female youth athletes exclusively and 20% (*n* = 13) worked with both male and females.

### 3.2. Perceptions of ERT in Youth

**Figure 1:**
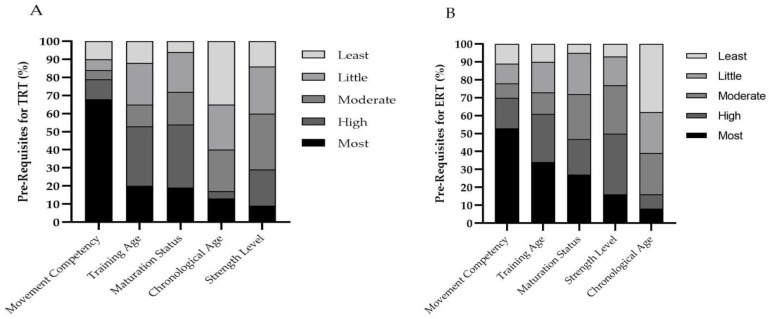
Coaches’ perceived importance of pre-requisites for (**A**) traditional (TRT) and (**B**) eccentric (ERT) resistance training in youth athletes.

The majority of coaches reported that they perceived both TRT and ERT to be important for youth athletes (Table 1). Whilst *trivial* differences were found in the combined agreement scores (TRT = 98% vs. ERT = 96%) a *small* difference existed between groups for the “strongly agree” category in which a greater number of coaches perceived TRT to be more important (PR = 1.13). Movement competency (68%, *n* = 44) was perceived as the most important pre-requisite prior to beginning TRT (Figure 1A) followed by the training age (20%, *n* = 13), maturation status (19%, *n* = 12), chronological age (13%, *n* = 8) and strength level (9%, *n* = 6). A similar order was reported for ERT (Figure 1B) in which movement competency (53%, *n* = 34) was followed by training age (34%, *n* = 22) and maturation status (27%, *n* = 17). However, strength level (16%, *n* = 10) and chronological age (8%, *n* = 5) were then subsequently noted. When “most-high” categories were combined, *small* differences were found between TRT compared to ERT for movement competency (79 vs. 70%, PR = 1.13) and maturation status (54 vs. 47%, PR = 1.15). Alternatively, a *small* difference was found between TRT compared to ERT for training age (53 vs. 61%, PR = 0.87). Additionally, a *moderate* difference was found between TRT compared to ERT for strength level (29 vs. 50%, PR = 0.58). The majority of coaches disagreed with all statements regarding the training responses to ERT in youth compared to adults (Table 2) for higher risk of injury (80%, *n* = 51), recovery time (78%, *n* = 50), muscle damage (69%, *n* = 44) and fatigue (67%, *n* = 43).

### 3.3. Implementation of ERT in Youth

**Figure 2:**
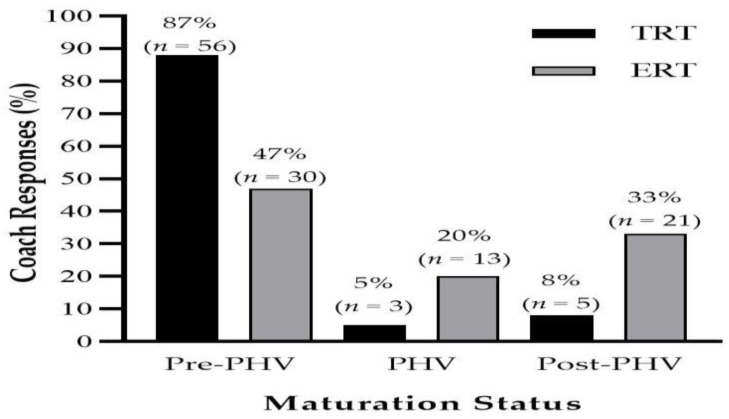
Maturation status at which coaches implement traditional (TRT) and eccentric (ERT) resistance training. PHV = peak height velocity.

**Figure 3:**
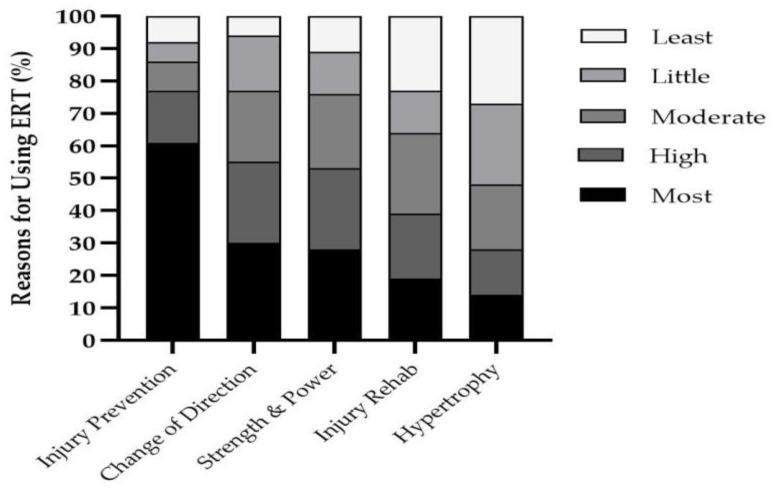
Reasons for utilizing eccentric resistance training (ERT) for youth athletes by coaches.

A *moderate* difference (PR = 1.85) was found between the number of coaches which included TRT during pre-peak height velocity (PHV) compared to ERT (Figure 2). Consequently, *very large* differences were found between TRT compared to ERT in which a greater number of coaches included ERT during PHV (PR = 0.25) and post-PHV (PR = 0.24). Coaches’ primary reason for utilizing ERT in youth (Figure 3) was for injury prevention purposes (61%, *n* = 39). This was then followed by change of direction (30%, *n* = 19), strength and power (28%, *n* = 18), injury rehabilitation (19%, *n* = 12) and muscle hypertrophy (14%, *n* = 9). Coaches reported that eccentric hamstrings training was the most important for youth athletes followed by isometric and concentric training (Table 3). Furthermore, ninety-one percent (91%) of coaches also stated that they prescribed the Nordic hamstrings exercise (NHE) to their youth athletes.

### 3.4. Training Prescription of ERT in Youth

**Figure 4:**
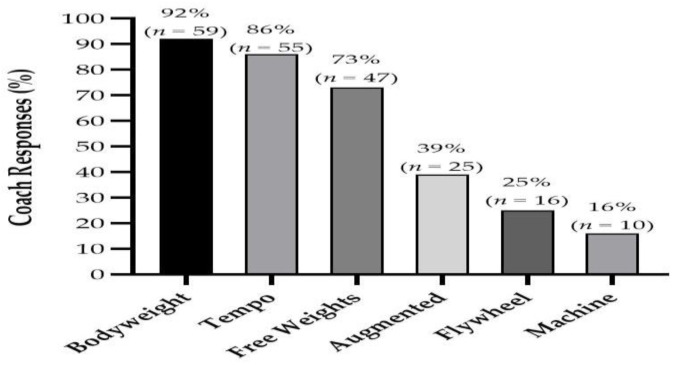
Reported eccentric resistance training modalities used by coaches for youth athletes.

**Figure 5:**
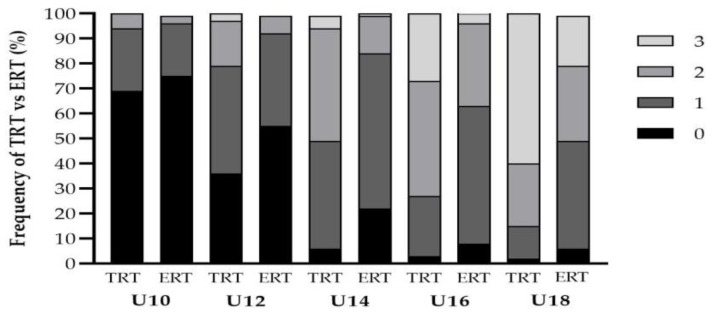
Weekly training frequency of traditional (TRT) and eccentric (ERT) resistance training across youth age groups.

**Figure 6:**
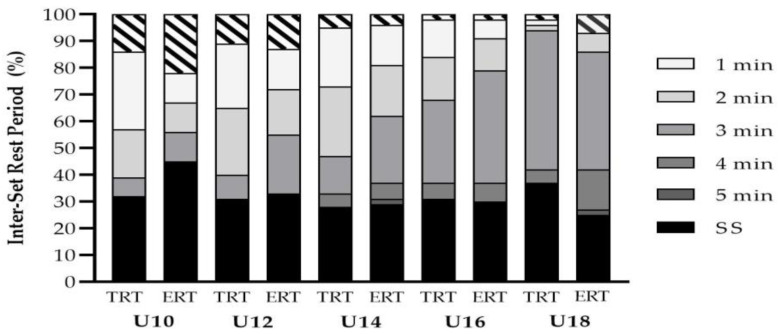
Inter-set rest period prescribed for traditional (TRT) and eccentric (ERT) resistance training across youth age groups. min = minute. SS = self-selected.

Coaches reported that body weight, tempo (e.g., greater emphasis on duration during the descent phase) and free weights training modalities were primarily used for ERT in youth (Figure 4). The weekly frequency of both TRT and ERT increased concurrently as age groups increased (Figure 5). However, a greater frequency of coaches prescribed TRT at all age groups compared to ERT with *large* differences between the groups found at U10 (6 vs. 3%, PR = 2.0), U12 (18 vs. 7%, PR = 2.57) and U14 (45 vs. 15%, PR = 3.0) age groups for two sessions per week. Additionally, *very large* to *large* differences were found at U14 (5 vs. 1%, PR = 5.0), U16 (27 vs. 5%, PR = 5.4) and U18 (60 vs. 20%, PR = 3.0) age groups for three sessions per week. With regards to the inter-set rest period (ISRP), the coaches reported that they mainly prescribed a self-selected ISRP for both RT methods at U10 (TRT = 32%, ERT = 46%), U12 (TRT = 31%, ERT = 33%) and U14 (TRT = 28%, ERT = 29%) age groups (Figure 6). However, a three-minute ISRP became more prevalent for both TRT or ERT at U16 (TRT = 31%, ERT = 42%) and U18 (TRT = 52%, ERT = 44%) age groups.

### 3.5. Barriers and Concerns for Utilizing ERT in Youth

**Figure 7:**
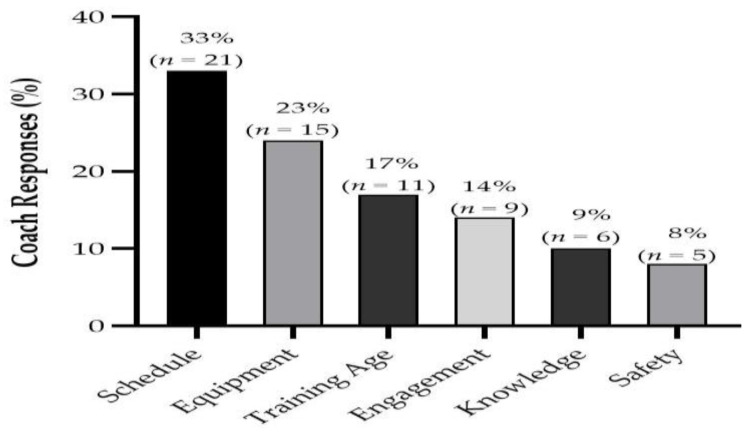
Coaches’ perceived barriers to the implementation of eccentric resistance training in youth athletes.

**Figure 8:**
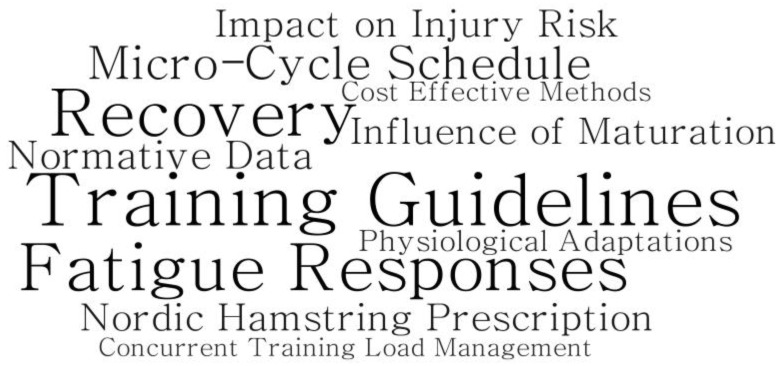
Word cloud presenting coaches’ perceived importance of areas for future research within ERT in youth athletes. This word cloud is based on the frequency of responses to the open questions. The more times a word is used the larger it appears.

Figure 7 shows the perceived barriers for the use of ERT in youth athletes. These main barriers were focused around logistical aspects such as training and match schedules as well as equipment required to perform ERT. With regards to future directions within ERT for youth, an array of areas was reported that practitioners felt required further information (Figure 8). These areas mainly included developing a better understanding of the training prescription for ERT in youth and how the maturation status may influence training adaptations, along with the fatigue and recovery responses to ERT too.

## 4. Discussion

The aim of this study was to investigate the perceptions and current practices of ERT for youth athletes by S&C coaches. To the authors’ knowledge, this is the first study to survey ERT in youth athletes, and to identify the current practices, perceptions and barriers that S&C coaches have regarding its use. Overall, coaches believed that ERT is important for youth athletes with training age, strength levels and the maturation status of the individual influencing its inclusion. There appeared to be a good understanding of the responses to eccentric exercise in youth with injury prevention the primary reason for the inclusion of ERT. Barriers for the implementation of ERT in youth were largely based around logistical factors with coaches also highlighting the need for further research into the different ERT methods for youth in order to provide further information on training guidelines.

Coaches agreed that the inclusion of both TRT and ERT is important for youth athletes. Although coaches believed that movement competency is the most important pre-requisite to begin either TRT or ERT, a greater proportion of responses for ERT highlighted the necessity of training age and strength levels. Evidence for why ERT requires a greater emphasis on these latter factors in youth is unclear. In adult athletes, previous research has found moderate to very high positive relationships between concentric and eccentric strength capabilities [47]. It is thus reasonable to presume that the consistent inclusion of dynamic RT exercises throughout youth will result in increases in eccentric as well as concentric force capabilities. However, responses to RT have been shown to be specific to the muscle action type trained [48]. Furthermore, greater levels of force are produced during maximal eccentric vs. concentric muscle actions [49]. Therefore, the delay of integrating ERT during youth may limit the development of eccentric strength. For instance, adolescent soccer players have been shown to have difficulty in reaching eccentric-overload during flywheel exercises [50]. The result of not developing eccentric strength qualities early on in youth may potentially impact the performance of sporting tasks. Indeed, eccentric knee extensor strength has been associated with greater deceleration ability in youth male soccer players [51]. Consequently, whilst future research should ascertain if a threshold of strength should exist prior to beginning ERT in youth, coaches should be mindful of the potential limitations in delaying its inclusion for tasks which require eccentric strength that are fundamental to athletic motor skill competencies (e.g., landing, change of direction).

Coaches demonstrated an awareness of the physiological responses to eccentric exercise in youth. For example, coaches largely disagreed that greater risks of muscle damage, fatigue and recovery would exist in youth compared to adults. Indeed, this is supported by current literature within the area among both males and females [22,23]. However, TRT was reported to mainly begin being prescribed during pre-PHV whilst the inclusion of ERT was more varied across maturation stages. The timing of when it is appropriate to begin ERT in youth is still unclear. Concerns for its inclusion too early in childhood could be due to the reported inefficiencies in male youth athletes (12.1 ± 1.1 yrs) in utilizing their stretch-shortening cycle (SSC) [52]. Indeed, the efficiency of the utilization of the SSC improves as the athlete matures [53]. However, positive responses to ERT have been shown following training interventions prescribing eccentric hamstrings training and flywheel inertia training (FIT) in pre-PHV athletes [24,30,31,32]. Additionally, post-PHV athletes have shown improvements in performance after completing such methods as well [25,26,27,28,33,54]. However, it should be acknowledged that further research is required to better understand how maturation may influence the responses to these methods in youth and the subsequent differences in training stimuli required as the athlete matures. Nevertheless, whilst some coaches may potentially favour a lower use of ERT during childhood and early adolescence, current literature suggests its inclusion can be considered an appropriate stimulus.

The primary reason for including ERT for youth athletes was for injury prevention purposes. Practitioners working with youth athletes have previously highlighted the requirement of lower-limb strength and eccentric hamstrings strength to prevent injuries [40]. Accordingly, nearly all coaches reported the importance of using eccentric muscle actions to develop hamstrings strength compared to concentric and isometric. Furthermore, 91% of our subjects reported that they prescribed the NHE. Whilst the reduction in hamstrings injuries in youth athletes via using the NHE is yet to be fully established, its use in adult athletes has been shown to reduce hamstrings injury risk by up to 51% [55]. The efficacy of such an approach during youth is becoming more salient with longitudinal analysis investigating injuries in youth practicing soccer and sprinting reporting hamstrings strains to be common during youth [56,57]. Furthermore, the use of ERT in youth may be beneficial for reducing the risk of injuries such as patella tendinopathy which is known to be impacted due to growth [58]. Indeed, following six weeks of flywheel training, an improved patella tendon condition was found in female youth athletes [59]. Moving forward, it is necessary to further investigate the impact of ERT on injury risk factors and the corresponding training guidelines that optimize this.

Coaches primarily used bodyweight and tempo training for ERT. Previously, the use of tempo training in youth male rugby union players (15.0 ± 0.9 yrs) was shown to enhance change of direction (COD) performance [7]. However, further research for its inclusion in youth is currently limited. Coaches increased both TRT and ERT frequencies in accordance with increases in age, although the frequency was consistently lower for ERT than TRT. The increase in RT frequency is in accordance with current RT guidelines in youth which suggest increases in weekly frequency in accordance with maturity status [60]. The lower frequency of ERT is likely expected considering that eccentric exercise has been shown to result in greater levels of muscle damage, fatigue, and time to recover than concentric training [12]. This approach is likely relevant for youth as well, since children and adolescents exhibit muscle damage after eccentric exercise, albeit to a lesser extent than their adult counterparts [22,23]. However, it should be noted that the aforementioned studies did not use well-trained youth athletes. Additionally, there was a tendency for self-selected rest periods to be preferred throughout U10 to U14 age groups. Such an approach is contrary to current evidence as previous research has reported the inability of less mature youth athletes to regulate their performance when using self-selected rest periods [61]. However, once athletes entered the U16 and U18 age groups, rest periods of three minutes were mainly prescribed, which may reflect a more specific training prescription approach once the athlete reaches post-PHV status. Indeed, as maturation increases, a longer recovery time is required to replenish energy resources [62].

The most frequently reported barriers for the inclusion of ERT in youth were focused around logistical aspects such as the training schedule and equipment. Previously, factors such as available time and equipment (e.g., budgetary constraints, minimal equipment and facilities available) have been reported to be important factors for the inclusion of injury prevention programme in youth [40]. Indeed, “cost effective” methods for ERT in youth were highlighted by coaches as an area for future research within ERT for youth in our study. Therefore, it is important for future research to identify ways in which ERT can be successfully integrated into training. Furthermore, coaches noted the need for further information on the practical methods of ERT in youth. Specifically, coaches commented that further information for aspects such as training guidelines for ERT is required as well as a better understanding of how maturation influences training adaptations. Accordingly, coaches also highlighted the need to understand the fatigue and recovery responses to ERT in youth as this is likely to affect how the micro-cycle is scheduled as well as the management of the training load. Subsequently, researchers can further investigate the areas highlighted here by S&C coaches in order to better inform their applied practices.

Despite our novel findings, our study is not without its limitations. For instance, the survey was potentially biased toward those coaches that actually currently use ERT with their youth athletes. Understanding the reasons for those coaches not using ERT among youth athletes could provide further clarification for this practice. Furthermore, most of the coaches were from the United Kingdom and therefore current practices presented in this study may not reflect those from other countries or regions which may not have access to the same extent of sport science literature. Unfortunately, our findings are mainly representative of the practices of youth male athletes. As previously noted, 2% of coaches worked only with female youth athletes and a further 20% worked with both male and females. It has been previously highlighted that the body of research relating to RT in female youth is substantially smaller than that in male youth and therefore requires further investigation [63]. The use of ERT in youth female athletes may be particularly necessary to understand, considering that low eccentric hamstrings strength is associated with landing mechanics that place the individual at greater risk of anterior cruciate ligament injury [64]. Therefore, further knowledge of how eccentric strength can be developed in female youth athletes and its relation to performance tasks and injury prevention is also necessary.

## 5. Conclusions

To the authors’ knowledge, this is the first study to survey the perceptions and practices of ERT in youth athletes. The findings from this study demonstrate that coaches agree that the use of ERT in youth is important and that they display a good awareness of the physiological responses to eccentric exercise in youth. Furthermore, it was apparent that injury prevention is the primary reason for the inclusion of ERT and that a focus on improving eccentric hamstrings strength is deemed necessary. Despite this, a large proportion of coaches reported to not begin ERT during pre-PHV. In light of the available research in this area, we would recommend that practitioners consider the adoption of ERT earlier on in youth. With regards to training prescription factors such as exercise selection, rest periods and training frequency appear to be operating on anecdotal information, beliefs or ERT research conducted in other populations. It is for this reason that the practices reported here should be interpreted with caution. Overall, based upon the received responses, it is evident that further research is required in order to provide coaches with ERT guidelines that enhance both performance and injury prevention aspects.

## Figures and Tables

**Table 1:** Coaches’ perceived importance of traditional (TRT) and eccentric (ERT) resistance training in youth athletes.

Training Type	Strongly Agree % (No.)	Agree % (No.)	Unsure % (No.)	Disagree % (No.)	Strongly Disagree % (No.)
TRT	76 (49)	22 (14)	0 (0)	2 (1)	0 (0)
ERT	67 (43)	29 (19)	2 (1)	2 (1)	0 (0)

**Table 2:** Coaches’ perceptions of responses to eccentric resistance training in youth compared to adults.

Training Response	Strongly Disagree % (No.)	Disagree % (No.)	Unsure % (No.)	Agree % (No.)	Strongly Agree % (No.)
Risk of Injury	22 (14)	58 (37)	17 (11)	3 (2)	0 (0)
Recovery Time	20 (13)	58 (37)	13 (8)	9 (6)	0 (0)
Muscle Damage	17 (11)	52 (33)	23 (15)	8 (5)	0 (0)
Fatigue	17 (11)	50 (32)	19 (12)	14 (9)	0 (0)

**Table 3:** Coaches’ perceived importance of muscle action type for hamstrings training in youth.

Muscle Action Type	High Importance % (No.)	Important % (No.)	Moderate % (No.)	Low Importance % (No.)	Not Important % (No.)
Eccentric	79 (51)	21 (13)	0 (0)	0 (0)	0 (0)
Isometric	27 (17)	52 (33)	19 (12)	2 (2)	0 (0)
Concentric	19 (12)	55 (35)	19 (12)	7 (5)	0 (0)

## Data Availability

All data is available within the manuscript.

## Supplementary Materials

Full questionnaire originally provided to all coaches. The following are available online at https://www.mdpi.com/2411-5142/6/1/21/s1.
