# Supplementary material for: Eccentric Resistance Training in Youth: A Survey of Perceptions and Current Practices by Strength and Conditioning Coaches
Source: J Funct Morphol Kinesiol. 2021 Feb 18;6(1):21. doi: 10.3390/jfmk6010021 (PMC8006255; doi:10.3390/jfmk6010021)
Supplement: Supplementary file 1 [file jfmk-06-00021-s001.pdf]

Lower-Body Eccentric Strength Training in Youth Team Sport Athletes: Current Perceptions and Practices of Youth S&C Coaches

Page 1: Introduction

You are being invited to take part in this study which aims to **examine the current perceptions and practices of lower-body eccentric strength training in youth team-sport athletes**. We hope to gain a better understanding of the use of eccentric strength training in youth compared to that of traditional strength training. This will include aspects such as training prescription, testing procedures and potential barriers to its inclusion.

To be eligible for this study:

- You must be over the age of 18.
- Currently working with youth team-sport athletes (18 years and under).

Participation in this study is **voluntary, and you have the right to stop answering the questions and withdraw your answers at any stage you wish**. We encourage you to answer all of the questions but any questions that you feel you are unable to answer then please leave blank. The survey should take approxiately 15-20 minutes of your time.

Ethical approval for this questionnaire has been provided by the [redacted] University Ethics Committee. All information collected in this questionnaire is done so anonymously and data will be stored in accordance with the Data Protection Act (1998). Should you wish to discuss any aspect of this questionnaire then you are able to contact the lead researcher [redacted].

By agreeing to take part in this survey you provide permission for your results you be used in an upcoming research publication presenting the findings from this survey. Should the results of this survey be submitted as a research manuscript an electronic copy will be kept in the repository at [redacted] University. Presentation of results in an academic journal, conference presentation or any other form of knowledge dissemination activity will not compromise the privacy of any involved participant.

We thank you for your time and effort in helping with this.

1. I provide my consent to participate in this survey (tick): ✱ *Required*

☐ YES ☐ NO

Page 2: Definitions

Eccentric Strength Training Definition:

Eccentric strength training (EST) is considered as using a load during the eccentric phase that is in excess of the concentric load (Wagle et al., 2017). The intensity of this stimulus can be submaximal or supramaximal (>100% muscle voluntary contraction). This may include modalities such as augmented eccentric loading, plyometrics, flywheel training and the nordic hamstring exercise.

Traditional Strength Training Definition:

Traditional strength training (TST) is considered as an emphasis on the loading the upward concentric phase of an exercise using resistance or body mass (Behm et al., 2017). This may include exercises such as squat, deadlift and leg press etc. in which the loading is prescribed as a % of concentric 1RM.

Page 3: Questions

2. To what extent do you agree that using traditional strength and eccentric strength training methods during youth is important for performance and injury prevention? ✱ *Required*

	Strongly Agree	Agree	Unsure	Disagree	Strongly Disagree
Traditional	☐	☐	☐	☐	☐
Eccentric	☐	☐	☐	☐	☐

3. Which of the following do you consider are pre-requisites prior to the inclusion of traditional (TST) and eccentric strength training (EST) in youth? Rank 1 (most important) to 5 (least important)

	**Traditional** ✱ *Required*					Eccentric ✱ *Required*				
	1	2	3	4	5	1	2	3	4	5
Chronological Age	☐	☐	☐	☐	☐	○	○	○	○	○
Growth Status	☐	☐	☐	☐	☐	○	○	○	○	○
Strength Level	☐	☐	☐	☐	☐	○	○	○	○	○
Resistance Training History	☐	☐	☐	☐	☐	○	○	○	○	○
Movement Competency	☐	☐	☐	☐	☐	○	○	○	○	○

4. In which chronological age group do you begin TST and EST with your youth athletes? ✱ *Required*

	< 9 years	10 years	11 years	12 years	13 years	14 years	15 years	16 years	17 years	18 years
Traditional	☐	☐	☐	☐	☐	☐	☐	☐	☐	☐
Eccentric	☐	☐	☐	☐	☐	☐	☐	☐	☐	☐

5. At which stage of growth do you begin including TST and EST with your youth athletes? ✱ *Required*

	Pre- Peak Pubertal Growth	Peak Pubertal Growth	Post-Peak Pubertal Growth
Traditional	☐	☐	☐
Eccentric	☐	☐	☐

6. What are your reasons for including ERT during youth? (List 1-5 in order of perceived importance; 1 being most important, 5 being least important) ✱ *Required*

	1	2	3	4	5
Strength and Power	☐	☐	☐	☐	☐
Muscle Hypertrophy	☐	☐	☐	☐	☐
Deceleration and Change of Direction	☐	☐	☐	☐	☐
Injury Prevention	☐	☐	☐	☐	☐
Injury Rehabilitation	☐	☐	☐	☐	☐

7. To what extent do you agree with the following statements? ✱ *Required*

	Strongly Agree	Agree	Unsure	Disagree	Strongly Dissagree
EST causes greater fatigue in youth compared to adults.	☐	☐	☐	☐	☐
EST causes greater muscle damage in youth compared to adults.	☐	☐	☐	☐	☐
Following EST, youth athletes are unable to recover as quickly as adults.	☐	☐	☐	☐	☐
EST is too much of an advanced resistance-training strategy for youth.	☐	☐	☐	☐	☐
Youth athletes are at a higher risk of injury than adults when performing EST.	☐	☐	☐	☐	☐

8. What types of EST exercises do you currently include with your youth athletes? (Select all that apply) ✱ *Required*

- ☐ Augmented Eccentric Loading
- ☐ Machine Exercises
- ☐ Free Weights (Dumbbell or Barbell)
- ☐ Body Weight Exercises
- ☐ Flywheel Inertia Training
- ☐ Tempo (descent phase emphasis)
- ☐ Other

8.a. If you selected Other, please specify:

9. To what extent do you agree with the following statement; ✱ *Required*

	Strongly Agree	Agree	Neither Agree or Disagree	Disagree	Strongly Disagree
Lower body eccentric strength is important for reducing injury risk during landing/cutting tasks in youth.	☐	☐	☐	☐	☐
Increasing force absorption qualities will enable youth athletes to better perform landing/cutting tasks.	☐	☐	☐	☐	☐

High levels of eccentric hamstrings strength should be developed in youth to help improve landing technique.	☐	☐	☐	☐	☐
High force absorption qualities during landing is needed prior to beginning plyometrics in youth.	☐	☐	☐	☐	☐

10. How many sessions per week do you schedule (in the youth age group you work with) TST and EST exercises?

	Traditional *Optional*				Eccentric *Optional*			
	0	1	2	3	0	1	2	3
< Under 9s	☐	☐	☐	☐	☐	☐	☐	☐
Under 10s	☐	☐	☐	☐	☐	☐	☐	☐
Under 11s	☐	☐	☐	☐	☐	☐	☐	☐
Under 12s	☐	☐	☐	☐	☐	☐	☐	☐
Under 13s	☐	☐	☐	☐	☐	☐	☐	☐
Under 14s	☐	☐	☐	☐	☐	☐	☐	☐
Under 15s	☐	☐	☐	☐	☐	☐	☐	☐
Under 16s	☐	☐	☐	☐	☐	☐	☐	☐
Under 17s	☐	☐	☐	☐	☐	☐	☐	☐
Under 18s	☐	☐	☐	☐	☐	☐	☐	☐

11. Within the training micro-cycle in which you have one match per week, when are eccentric resistance focused exercises scheduled?
✱ *Required*

- ○ Match Day +1
- ○ Match Day +2
- ○ Match Day +3
- ○ Match Day - 3
- ○ Match Day - 2
- ○ Match Day -1
- ○ Not applicable

12. What inter-set rest intervals do/would you use (in the age groups you work with) when prescribing TST and EST in youth athletes?

	Traditional *Optional*							Eccentric *Optional*						
	<1 minute	1 min	2 mins	3 mins	4 mins	5 mins	Self Selected	<1 minute	1 min	2 mins	3 mins	4 mins	5 mins	Self Selected
< Under 9s	○	○	○	○	○	○	○	☐	☐	☐	☐	☐	☐	☐
Under 10s	○	○	○	○	○	○	○	☐	☐	☐	☐	☐	☐	☐
Under 11s	○	○	○	○	○	○	○	☐	☐	☐	☐	☐	☐	☐

Under 12s														
Under 13s														
Under 14s														
Under 15s														
Under 16s														
Under 17s														
Under 18s														

13. What level of importance do you place on using the following muscle action types for developing hamstring strength throughout youth for your athletes? ✱ *Required*

Please don't select more than 1 answer(s) per row.

Please select at least 1 answer(s).

	High Importance	Important	Moderate Importance	Low Importance	Not Important
Concentric					
Eccentric					
Isometric					

14. Which hamstrings exercises do you include for your youth athletes? (Select all that apply) ✱ *Required*

- ☐ Unilateral Romanian Deadlift
- ☐ Isometric Holds
- ☐ Sliders
- ☐ Good Mornings
- ☐ Straight Knee Bridge
- ☐ Slide Leg Curl
- ☐ Prone Leg Curl
- ☐ Hip Extensions
- ☐ Sprinting
- ☐ Other

14.a. If you selected Other, please specify:

15. Do you prescribe the Nordic Hamstring Exercise (NHE) or a variation of the NHE within your training programme for your youth athletes? ✱ *Required*

- ◯ YES
- ◯ NO

16. **If YES to question 15;** What number of repetitions do you use (in the age groups you work) for the NHE in your youth athletes?

➕ More info

	Age Group										Additional Comments
	<Under 9s	Under 10s	Under 11s	Under 12s	Under 13s	Under 14s	Under 15s	Under 16s	Under 17s	Under 18s	
1-5 reps	☐	☐	☐	☐	☐	☐	☐	☐	☐	☐	
6-10 reps	☐	☐	☐	☐	☐	☐	☐	☐	☐	☐	
11-15 reps	☐	☐	☐	☐	☐	☐	☐	☐	☐	☐	
Reps To Failure	☐	☐	☐	☐	☐	☐	☐	☐	☐	☐	

17. **If YES to question 15**; What number of sets do you use (in the age groups you work) for the NHE with your youth athletes?

	Age Groups										Additional Comments
	<Under 9s	Under 10s	Under 11s	Under 12s	Under 13s	Under 14s	Under 15s	Under 16s	Under 17s	Under 18s	
1 set	☐	☐	☐	☐	☐	☐	☐	☐	☐	☐	
2 sets	☐	☐	☐	☐	☐	☐	☐	☐	☐	☐	
3 sets	☐	☐	☐	☐	☐	☐	☐	☐	☐	☐	
4 sets	☐	☐	☐	☐	☐	☐	☐	☐	☐	☐	
>5 sets	☐	☐	☐	☐	☐	☐	☐	☐	☐	☐	

18. When do you schedule the NHE, or other eccentric injury prevention exercises, within the training session? ✱ *Required*

- ○ Pre Session
- ○ During Session
- ○ Post Session
- ○ Independent Injury Prevention Session

19. What tests do you use to evaluate hamstrings strength in your youth athletes? (Select all that apply) ✱ *Required*

- ☐ NONE
- ☐ Isokinetic Dynamometer
- ☐ Isometric Bridge Hold
- ☐ Nordic (e.g. Nordbord, Hamstring Solo)
- ☐ Handheld Dyamometer Testing
- ☐ Supine Isometric Hamstring (Force Plate)
- ☐ Other

19.a. If you selected Other, please specify:

20. Which of the following measures are of interest when you test hamstring strength in your youth athletes? (Select all that apply) ✱ *Required*

- ☐ NONE
- ☐ Peak/Average Force
- ☐ Peak/Average Torque
- ☐ Inter-Limb Asymmetries
- ☐ Breakpoint Angle (Nordic)
- ☐ Hamstring:Quadricep Ratio
- ☐ Angle of Peak Torque (Functional Range)
- ☐ Duration of Hold (Isometric)
- ☐ Other

20.a. If you selected Other, please specify:

21. What sources currently inform your prescription/approach to ERT in youth? ✱ *Required*

- ☐ Personal Knowledge and Experience
- ☐ Sporting National Governing Body
- ☐ Internal Club/Organisation Research

- ☐ Published Research
- ☐ S&C Colleagues/Network
- ☐ Additional Courses/Qualifications/CPD Events
- ☐ Other

21.a. If you selected Other, please specify:

22. What barriers do you feel you face when implementing EST with your youth athletes? (Select all that apply) ✱ *Required*

- ☐ NONE
- ☐ Lack Of Equipment
- ☐ Training Age
- ☐ Time
- ☐ Knowledge and understanding of appropriate ERT methods
- ☐ Athlete Engagement
- ☐ Education
- ☐ Safety Concerns
- ☐ Schedule
- ☐ Training Prioritisation
- ☐ Other

22.a. If you selected Other, please specify:

23. How confident are you currently when implementing EST modalities with your youth athletes? ✱ *Required*

- ○ Very Confident
- ○ Confident
- ○ Unsure
- ○ Not Confident
- ○ Wouldn't Implement
- ○ Other

23.a. If you selected Other, please specify:

24. Do you have any concerns when utilising EST with youth? (Please list answers in bullet points) ✱ *Required*

Your answer should be no more than 300 characters long.

25. Are there any issues/areas surrounding EST in youth that you feel require greater information moving forward? (Please list answers in bullet points) ✱ *Required*

Your answer should be no more than 300 characters long.

Page 4: Personal Information

26. Sport you are currently working in: ✱ *Required*

27. Club/Organisation Level: ✱ *Required*

- ☐ Professional
- ☐ Semi-Professional
- ☐ Amateur
- ☐ School/College

28. Sex of youth team sport athletes you are currently working with: ✱ *Required*

○ Male ○ Female ○ Both

29. Country: ✱ *Required*

30. Name (Optional) :

31. Contact Email (Optional):

Page 5: Thank You

Thank you for taking the time to answer these questions. If you provided your name and email address above then we will we contact you via email with the results from the survey in due course.
